# Effect of Narcissistic Personality on Entrepreneurial Intention Among College Students: Mediation Role of Entrepreneurial Self-Efficacy

**DOI:** 10.3389/fpsyg.2021.774510

**Published:** 2022-02-07

**Authors:** Sun-Yu Gao, Jianhao Huang

**Affiliations:** ^1^Dhurakij Pundit University, Bangkok, Thailand; ^2^Hainan Technology and Business College, Hainan, China

**Keywords:** entrepreneurial intention (EI), narcissistic personality, entrepreneurial self-efficacy (ESE), college students, mediation role

## Abstract

Exploring the factors influencing entrepreneurial intention is crucial to entrepreneurial practice and education. For a comprehensive understanding of the influence of narcissistic personality on entrepreneurial intention, this study analyzed the relationship between narcissistic personality, entrepreneurial self-efficacy, and entrepreneurial intention in college students sampled from three higher vocational colleges in Beijing, China. A total of 252 valid questionnaires were collected. The results show that the narcissistic personality of the college students has a significant positive effect on entrepreneurial intention and entrepreneurial self-efficacy. Entrepreneurial self-efficacy of the college students has a significant positive effect on entrepreneurial intention and plays a partial mediation role in the relationship between narcissistic personality and entrepreneurial intention. Thus, the study results provide some reference for further improving entrepreneurial practice and education.

## Introduction

With rapid global advancement of technology, entrepreneurial abilities are gaining increasing prominence. Work of domestic and foreign researchers on entrepreneurial intention has borne fruit (Wang et al., 2020; Huang et al., 2021; Hoang and Le, 2021; Shahin et al., 2021). Other studies have shown that entrepreneurial intention of college students affects their future entrepreneurial behaviors (Boubker et al., 2021; Elnadi and Gheith, 2021). Thus, the key factors and influencing mechanisms that affect entrepreneurial intentions must be explored.

Some specific personality traits are prerequisites for entrepreneurship and can influence entrepreneurial intentions (Utsch and Rauch, 2000). Narcissistic personality, one such personality trait, describes the structure of behavior patterns in an individual’s life. Koh (1996) defined the following prerequisites for narcissistic personality: internal control, need for achievement, moderate levels of risk-taking, innovation, great self-confidence, and high tolerance of ambiguity. Research has shown that narcissistic personality affects entrepreneurial intention (Mathieu and St-Jean, 2013; Wu et al., 2019a; McLarty et al., 2021), and this impact has been the focus of current research. However, results of empirical studies are scarce. Therefore, investigating this topic further to obtain additional evidence to remedy deficiencies in past research is worthwhile.

In addition, being an important topic in entrepreneurship-related research, entrepreneurial self-efficacy has attracted the attention of many researchers (Chen et al., 1998; DeNoble et al., 2007; Borchers and Park, 2010). Entrepreneurial self-efficacy is a concept derived from self-efficacy. Some studies have suggested that entrepreneurial self-efficacy has a significant positive effect on entrepreneurial intention (Chen et al., 1998; DeNoble et al., 2007; Borchers and Park, 2010). Some have also found that narcissistic personality significantly and positively affects entrepreneurial self-efficacy (Wu et al., 2019a; Al-Ghazali and Afsar, 2020; Hegde and Shetty, 2020). Moreover, entrepreneurial self-efficacy often plays a mediation role in entrepreneurial intention-related studies. For example, Zhao et al. (2005) found that entrepreneurial self-efficacy mediates the relationship between entrepreneurial experience and entrepreneurial intention. Prabhu et al. (2012) found that entrepreneurial self-efficacy acts as a mediator in the relationship between positive personality and entrepreneurial intentions. Wu et al. (2019a) found that entrepreneurial self-efficacy plays a mediation role in the relationship between the narcissistic dimension of the dark triad and entrepreneurial intention. Therefore, we believe that entrepreneurial self-efficacy also plays a mediation role in the relationship between narcissistic personality and entrepreneurial intentions.

The aims of the study are twofold: first, most studies on narcissistic personality and entrepreneurial intention are based on samples from the United States (O’Reilly et al., 2014; Hmieleski and Lerner, 2016). Therefore, we used Chinese college students as the study sample to compensate for the shortcomings of past studies. Second, among the factors related to entrepreneurial intention, personality traits are critical predictors of entrepreneurial intention. Few researchers conducted research on college students in China’s vocational colleges. However, a more diverse sample must test the association between narcissistic personality and entrepreneurial intentions. Third, the current research applies narcissistic personality, entrepreneurial self-efficacy, and entrepreneurial intention to the theory of self-efficacy to understand the mediating role of entrepreneurial self-efficacy in the relationship between narcissistic personality and entrepreneurial intention. However, the influence mechanism of this relationship is still unclear. Therefore, the current study uses Chinese vocational college students as the research sample, narcissistic personality as the independent variable, entrepreneurial self-efficacy as the intermediary variable, and entrepreneurial intention as the dependent variable to explore the three aspects of the narcissistic personality entrepreneurial self-efficacy, and entrepreneurial intention. The inter-influence mechanism provides a new direction for improving college students’ entrepreneurial intentions and further enriches self-efficacy theory.

The research questions of the current study are as follows:

1. Does the narcissistic personality of college students in Beijing, China affect their entrepreneurial intentions?
2. Does the narcissistic personality of college students in Beijing, China affect entrepreneurial self-efficacy?
3. Does the entrepreneurial self-efficacy of college students in Beijing, China affect their entrepreneurial intentions?
4. Does the entrepreneurial self-efficacy of college students in Beijing, China play a mediating role in the influence of narcissistic personality on entrepreneurial intention?

## Literature Review and Hypothesis Development

### Self-Efficacy Theory

Based on self-efficacy theory, we seek to understand the influence mechanism among narcissistic personality, entrepreneurial self-efficacy, and entrepreneurial intention. This theory states that self-efficacy is an individual’s judgment, beliefs, or feelings about their ability to complete an activity at a certain level. Self-efficacy is related to an individual’s ability level and personality traits; however, it does not represent their true ability level (Bandura, 1997). Narcissistic personality is an individual trait (Brown et al., 2009). Mature narcissism in good shape produces humorous and creative behaviors; thus, narcissistic traits can be beneficial for the career of narcissists (Furnham and Crump, 2014). On the other hand, the higher the degree of entrepreneurial self-efficacy of an individual, the higher their entrepreneurial intention and the greater their chance of starting their own businesses (Boyd and Vozikis, 1994; Trevelyan, 2011).

### Narcissistic Personality and Entrepreneurial Intention

Narcissism is not necessarily pathological, but a separate chronological order, from infancy to adulthood, exists (Kohut, 1966). Narcissists tend to glorify themselves, displaying overconfidence, capriciousness, high-level histrionic personality, and intense aggression, but they are sensitive and anxious and have a strong sense of insecurity (Wink and Donahue, 1997). Moreover, some researchers have argued that narcissistic personality actually combines cognition, emotion, and behavior, jointly conveying an exaggerated, crucial, and unique self-concept (Rhodewalt and Eddings, 2002). Pincus and Lukowitsky (2010) considered narcissism as an aggressive, arrogant, and self-righteous personality trait. Bender (2012) suggested that narcissistic personality forms a part of our mental development and individual personality. Dhillon (2019) has put forward a fascinating view that narcissism refers to excessive admiration and interest in oneself and one’s appearance. In the current study, we adopted the definition of Dhillon (2019) and defined narcissistic personality as having or showing an extreme interest in or admiration of oneself and one’s physical appearance.

Some college students have a strong desire to start their own business, and some even put their ideas into practice while being still in school. Some differences can be observed in the personalities between these students and other students who seek employment after graduation. They are self-confident, like to try new things, and are ready to take risks (Mathieu and St-Jean, 2013). Based on a sample of respondents from high-tech companies in the United States, a relevant study found that narcissistic personality coincides with some characteristics of leaders. A person with narcissistic personality is generally dominant and has a strong sense of control and self-awareness (O’Reilly et al., 2014). A study (Hmieleski and Lerner, 2016) showed that narcissistic personality significantly influences entrepreneurs’ establishing their businesses and that people with high narcissistic personalities can live in the now and can quickly become leaders by starting a business. Narcissists have a higher drive to pursue, constantly seek advancement, and exhibit better decision-making ability (Zia et al., 2020). Consequently, we proposed hypothesis H1 of this study.

H1: College students’ narcissistic personality significantly and positively affects their entrepreneurial intention.

### Narcissistic Personality and Entrepreneurial Self-Efficacy

Entrepreneurial self-efficacy is the strength of an individual’s belief that they can successfully play different entrepreneurial roles and start their businesses (Boyd and Vozikis, 1994; McGee et al., 2009). It is also an individual’s belief in their ability to achieve goals, control positive and negative cognition when starting a business (Drnovšek et al., 2010), and perform new tasks successfully. It also refers to their expectations for establishing a new business (Pihie and Bagheri, 2013). This study defined entrepreneurial self-efficacy as the degree of an entrepreneur’s confidence and expectation that they can start a business to control the events in their lives after they knew their abilities and that they will be able to succeed in their ventures.

People with high narcissistic personalities tend to perform positive self-evaluations because they believe that they will achieve positive results, which in turn affects entrepreneurial self-efficacy (Campbell et al., 2004). These people generally have a strong motivation to pursue goals, are eager to seek attention and improve themselves, and are good at seizing opportunities (Forsyth et al., 2012; Do and Dadvari, 2017). They also believe they will be able to encounter obstacles, setbacks, and failures of starting businesses better than others (Mathieu and St-Jean, 2013). A study conducted by Brookes (2015) among psychology college students reported that people with high narcissism are more confident about achieving their goals, and the higher narcissistic personalities, the higher entrepreneurial self-efficacy. Some empirical studies have suggested that narcissistic personality has a significant positive impact on entrepreneurial self-efficacy (Al-Ghazali and Afsar, 2020; Hegde and Shetty, 2020). Thus, we proposed hypothesis H2 of this study.

H2: College students’ narcissistic personality significantly and positively affects their entrepreneurial self-efficacy.

### Entrepreneurial Self-Efficacy and Entrepreneurial Intention

Beck and Ajzen (1991) argued that entrepreneurial intention can be a plan to start a new business, an intention to predict future personal behavior and organizational outcomes, and an entrepreneurial belief that they intend to create a company or organization during the preparation period before starting a business (Souitaris et al., 2007). A person with thoughts and attitudes about the desire to own their business or establish a new business is believed to have entrepreneurial intention, which is a necessary core to understand the entrepreneurial process (Krueger and Carsrud, 1993). People more inclined to start businesses have a greater desire for achievement and greater self-confidence (Koh, 1996). Entrepreneurial activity involves a series of planned ideas and behaviors; therefore, an entrepreneur’s ideas and intentions are the basis for creating a new business and the starting point of the entrepreneurial process (Krueger et al., 2000). This study defines entrepreneurial intention as a person’s idea that they are prepared to start a business and have a plan to do so.

Entrepreneurial self-efficacy largely influences an individual’s intentions and behaviors of becoming an entrepreneur; their efforts to establish a new business; and perseverance in the face of new things, changes, and challenges in the process of establishing a new business (Trevelyan, 2011). Several researchers have found that entrepreneurial self-efficacy positively affects entrepreneurial intention. For example, in college students from Malaysia, Pihie and Bagheri (2013) found that students’ self-efficacy has a significant positive impact on their entrepreneurial intention. Liu et al. (2019) found that entrepreneurial self-efficacy has a significant positive effect on Entrepreneurial Intention in Chinese college students. A study conducted by Aima et al. (2020) among college students in Indonesia found that entrepreneurial self-efficacy has a significant positive impact on entrepreneurial intention. Through a sample survey of college students, Neneh (2020) found that entrepreneurial self-efficacy positively influences entrepreneurial intention. Results of the current study show that college students’ entrepreneurial self-efficacy significantly and positively affects entrepreneurial intention. Therefore, we proposed hypothesis H3 of this study.

H3: College students’ entrepreneurial self-efficacy significantly and positively affects their entrepreneurial intention.

### The Mediation Role of Entrepreneurial Self-Efficacy in the Relationship Between Narcissistic Personality and Entrepreneurial Intention

Based on the analysis of previous studies, we found that entrepreneurial self-efficacy often plays a mediation role in the relationship between individuals’ cognitive abilities. In master degree candidates from five Chinese universities, Zhao et al. (2005) found that entrepreneurial self-efficacy acts as a mediator in the effect of entrepreneurial experience on entrepreneurial intention and that of risk intention on entrepreneurial intention. Through a study on graduate and undergraduate students from four universities in China, Finland, Russia, and the United States, Prabhu et al. (2012) found that entrepreneurial self-efficacy mediates the relationship between proactive personality and entrepreneurial intention.

On combining hypotheses H1 and H3 and the results of the aforementioned analysis, we found that narcissistic personality can not only directly influence entrepreneurial intention but also indirectly affect it through entrepreneurial self-efficacy. Specifically, people with high narcissistic personalities will have higher entrepreneurial intentions because their personality traits have a higher drive to pursue and constantly seek advancement and they exhibit better decision-making ability (Zia et al., 2020). The higher entrepreneurial self-efficacy of a person, the higher their entrepreneurial intention and the higher odds of their starting their businesses (Boyd and Vozikis, 1994). By investigating Chinese college students, some researchers found that entrepreneurial self-efficacy mediates the relationship between narcissism and entrepreneurial intention (Wu et al., 2019a). Therefore, individuals with high levels of narcissistic personality tend to have higher entrepreneurial self-efficacy in the entrepreneurial process, which may further spawn entrepreneurial intention. Therefore, we proposed hypothesis H4 of this study.

H4: College students’ entrepreneurial self-efficacy plays a mediation role in the relationship between their narcissistic personality and entrepreneurial intention.

## Methods

### Research Framework

**FIGURE 1:**
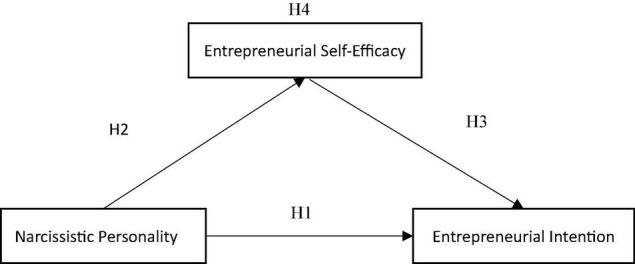
Research framework.

In this study, college students’ narcissistic personality, entrepreneurial intention, and entrepreneurial self-efficacy were used as independent, dependent, and mediating variables, respectively. Based on the research hypotheses, we proposed the research framework (Figure 1).

### Participants and Procedures

The study was divided into pre-test and formal phases. Complete data were tested and retrieved twice. Questionnaires were distributed to different participants.

### Pilot-Test Sample Used in the Study

The pre-test questionnaire was distributed on September 3, 2020 and returned on September 21, 2020. Using purposive sampling, we circulated 85 questionnaires to 85 college students in a higher vocational college in Beijing. Of them, 80 questionnaires were valid, so the questionnaire efficiency was 94.1%. After analyzing the reliability and validity of the questionnaires’ content, we deleted some items, revised the initial questionnaires, and finalized the official ones.

### Formal Sample Used in the Study

The formal questionnaire was distributed on October 4, 2020 and returned on October 25, 2020. Using purposive sampling, we conducted a questionnaire survey of college students in three higher vocational colleges in Beijing; these institutions are considered models of entrepreneurship education. We followed Ghiselli et al. (1981)’s proposed sampling criteria: if the study involves the use of scales, the study sample size should be at least 10 times the total number of questions. According to these criteria, the three scales used in this study had 25 questions and over 250 items of data that should be validly collected. The results of Israel (1992), the formula for calculating the sample size: Sample size = *z*^2^ × *p*(1 − *p*)/*e*^2^/1 + [*z*^2^ × *p*(1 − *p*)/*e*^2^N], *z* = 1.65, *N* = 15300, *p* = 0.5, *e*^2^ = 0.0025, The sample size of the study is approximately 268. It was expected that 300 questionnaires will be distributed. According to the proportion, 30, 30, and 40% of college students in each of the three higher vocational colleges were selected separately as respondents. We circulated the questionnaires on Wenjuanxing, an online questionnaire platform, and received 260 questionnaires. Finally, a total of 252 valid questionnaires were obtained. The effective recovery was 97%, which met the sampling criteria. We set two background variables, gender and grade, for the basic information of the students. Gender was categorized as male and female, and grade was classified as freshman, second-year university, and junior. Because senior students were on placement, they were not included as the study sample. In total, the study had 199 men (79% of the sum total) and 53 women (21% of the sum total). Overall, there were 136 freshmen (54% of the sum total), 61 second-year universities (24.2% of the sum total), and 55 juniors (21.8% of the sum total).

### Test Process

This study was carried out in accordance with the commendations of Hainan Technology and Business College. Before the test, the teachers of the respondents were informed of the study intention. With their assistance, we asked students to fill out the questionnaire in a uniform manner. To ensure the objectivity and authenticity of the questionnaire data, we explained the confidentiality of the questionnaire results and the study purpose to the respondents.

## Measures

The questionnaire used in the present study consisted of 25 items: 2 items measuring basic information about the respondents, 13 items of the narcissistic personality scale, 4 items of the entrepreneurial self-efficacy scale, and 6 items of the entrepreneurial intentions scale.

### Narcissistic Personality Scale

This study used the narcissistic personality Inventory-16 of Ames et al. (2006), which has 16 one-dimensional questions. We conducted an exploratory factor analysis of the pre-test results, eliminated questions with factor loadings of <0.4, and finalized 13 questions. According to the results, KMO was 0.851; factor loadings was 0.626–0.813, indicating good validity; and Cronbach’s α value was 0.913, indicating good reliability.

### Entrepreneurial Self-Efficacy Scale

This study used the entrepreneurial self-efficacy scale of Krueger et al. (2000), which has 4 one-dimensional questions. We conducted an exploratory factor analysis of the pre-test results. According to the analysis results, KMO was 0.814; factor loadings was 0.852–0.901, indicating good validity; and Cronbach’s α value was 0.900, indicating good reliability.

### Entrepreneurial Intention Scale

This study used the entrepreneurial intention scale of Liñán and Chen (2009), which has 6 one-dimensional questions. We conducted an exploratory factor analysis of the pre-test results. The results showed KMO was 0.832; factor loadings was 0.645–0.890, indicating good validity; and Cronbach’s α value was 0.895, indicating good reliability.

## Data Analysis

We used SPSS software was used to test the common method bias of the narcissistic personality scale, entrepreneurial self-efficacy scale, and entrepreneurial intentions scale. Then, we explored the relationship between these three main variables in SPSS through correlation analysis. Finally, we explored the specific relationship between pairs of the three variables and examined the mediation role of entrepreneurial self-efficacy in the impact of narcissistic personality on entrepreneurial intention.

### Common Method Bias

Harman’s single-factor test was performed to test common method bias based on the results of the formal questionnaire Harman’s single-factor (Podsakoff and Organ, 1986). The result meets the Common method bias test standard proposed by Hair et al. (1998). There are 4 factors with feature values greater than 1 extracted. The explanatory variance of the first factor is 42.539%, which is lower than the reference value is 50%. We can conclude that no Common method bias problem exists in the current study’s data.

## Results

### Descriptive Statistics and Correlation Analysis

Table 1 presents the descriptive statistics and correlations of the variables. According to the results, college students’ “NP” mean was 3.041, their “ESE” mean was 3.357, and their “EI” mean was 3.433. The level of narcissistic personality, entrepreneurial self-efficacy, and entrepreneurial intention of the college students was above average.

**TABLE 1:** Descriptive analysis and correlation analysis of the variables.

Variable	M	SD	NPI	ESE	EI
NP	3.041	0.700	1		
ESE	3.571	0.620	0.632***	1	
EI	3.433	0.700	0.544***	0.688***	1

***p < 0.001.

*NP, Narcissistic Personality; ESE, Entrepreneurial Self-Efficacy; EI, Entrepreneurial Intention.*

A significant positive correlation was observed between college students’ narcissistic personality and their entrepreneurial self-efficacy (*r* = 0.632, *p* < 0.001). Narcissistic personality was significantly and positively correlated to entrepreneurial intention among the college students (*r* = 0.544, *p* < 0.001). Entrepreneurial self-efficacy and entrepreneurial intention of college students were significantly and positively correlated (*r* = 0.688, *p* < 0.001). The correlation coefficients between the variables ranged from 0.544 to 0.688, with no high correlation and no serious collinearity. Details are given in Table 1.

### Regression Analysis

Empirical studies have shown that male college students’ entrepreneurial intention is considerably higher than their female counterparts’ (Yukongdi and Lopa, 2017; Molino et al., 2018; Jena, 2020); entrepreneurial intention of second-year universities is considerably higher than that of juniors and freshmen (Wang and Huang, 2019). The results of t-check and ANOVA check showed a large gender gap in terms of entrepreneurial intention (*t* = 4.764, *p* < 0.001), that is, the entrepreneurial intention of male students was higher than that of female students. Moreover, the results revealed a large grade gap (*F* = 290.200, *p* < 0.001), that is, the entrepreneurial intention of freshmen was higher than that of juniors, and the entrepreneurial intention of second-year universities was higher than that of juniors and freshmen. Therefore, this study included the demographic variables of gender and grade in the hierarchical regression analysis.

This study examined the mediating effect of college students’ entrepreneurial self-efficacy on the relationship between narcissistic personality and entrepreneurial intention based on the premise that the effects of demographic variables, that is, gender and grade, are controlled. As shown in Table 2, the narcissistic personality of college students significantly and positively impacted entrepreneurial intention (β = 0.234, *t* = 6.750, *p* < 0.001) in Model 1, and therefore, hypothesis H1 checks out. The narcissistic personality of college students significantly and positively affected entrepreneurial self-efficacy (β = 0.483, *t* = 9.880, *p* < 0.001) in Model 2; therefore, hypothesis H2 is valid. In Model 3, after adding the mediating variable entrepreneurial self-efficacy, college students’ narcissistic personality had a significant positive effect on entrepreneurial intention (β = 0.114, *t* = 2.975, *p* < 0.01) and their entrepreneurial self-efficacy significantly and positively influenced entrepreneurial intention (β = 0.247, *t* = 5.846, *p* < 0.001), and hence, hypothesis H3 checks out. The β value for the effect of the narcissistic personality of college students on entrepreneurial intention decreased from 0.234, up to the significant level, in Model 1 to 0.114, up to the significant level, in Model 3. Thus, entrepreneurial self-efficacy plays a partial mediation role in the effect of narcissistic personalities on entrepreneurial intention in college students, and so, hypothesis H4 is valid. Furthermore, this study used the Sobel test to verify the mediating effect and used unstandardized regression coefficients and standard errors to calculate it. The formulae for Sobel test which is *z*-value = *a* × *b*/SQRT(*b*^2^ × *s*_a_^2^ + *a*^2^ × *s*_b_^2^), a = raw (unstandardized) regression coefficient for the association between the Narcissistic Personality and the Entrepreneurial Self-Efficacy. *s*_a_ = standard error of *a*. *b* = raw coefficient for the association between the Entrepreneurial Self-Efficacy and the Entrepreneurial Intention (when the Narcissistic Personality is also a predictor of the Entrepreneurial Intention). *s*_b_ = standard error of *b*. *Z* value of >1.96 represents a significant mediating effect (Sobel, 1982), and the present study showed that entrepreneurial self-efficacy (*Z* = 9.714, *p* < 0.001) has a significant mediating effect. In Model 3, the VIF was <10 and no covariance problem was noted. Details are given in Table 2.

**TABLE 2:** Test of the mediation role of entrepreneurial self-efficacy in the effect of narcissistic personality on entrepreneurial intention.

Variable	Model 1		Model 2		Model 3		
	EI		ESE		EI		
	β	*t*	β	*t*	β	*t*	VIF
Male Students	0.081	2.486*	0.012	0.259	0.078	2.551*	1.071
Freshmen	0.567	13.750***	0.209	3.582***	0.516	12.979***	1.815
Second-year universities	0.915	20.840***	0.456	7.352***	0.803	17.627***	2.384
NP	0.234	6.750***	0.483	9.880***	0.114	2.975**	1.700
ESE					0.247	5.846***	2.061
R^2^	0.756		0.515		0.786		
Adj R^2^	0.752		0.507		0.782		
*F*	191.701***		65.497***		180.795***		

*, p < 0.05; **, p < 0.01; ***, p < 0.001.

*β is the standardized regression coefficient.*

*Gender and grade are dummy variables, in which male students are the experimental group and female students are the reference group, and freshmen and second-year universities form the experimental group and junior students are the reference group, respectively.*

*NP, Narcissistic Personality; ESE, Entrepreneurial Self-Efficacy; EI, Entrepreneurial Intention.*

### Revalidation of the Mediating Role of Entrepreneurial Self-Efficacy

This study further uses PROCESS macro model 4 to test the mediating role of entrepreneurial self-efficacy in the relationship between narcissistic personality and entrepreneurial intention. Controlling gender and grade as covariates, the results show that narcissistic personality significantly predicts entrepreneurial intention (*B* = 0.235, *t* = 6.750, *p* < 0.001). After adding entrepreneurial self-efficacy as an intermediary variable, narcissistic personality is still an important predictor of entrepreneurial intention (*B* = 0.115, *t* = 2.975, *p* < 0.01). In addition, narcissistic personality positively predicts entrepreneurial self-efficacy (*B* = 0.431, *t* = 9.880, *p* < 0.001), entrepreneurial self-efficacy positively predicts entrepreneurial intention (*B* = 0.279, *t* = 5.846, *p* < 0.001), see Table 3 for details. In addition, according to the bootstrap test, entrepreneurial self-efficacy has a significant indirect effect (*CI* = 0.067–0.184, effect size = 0.120). Thus, entrepreneurial self-efficacy plays a partial mediation role in the effect of narcissistic personalities on entrepreneurial intention in college students.

**TABLE 3:** Testing the mediation model of entrepreneurial self-efficacy.

Variables	Model1			Model2			Model3		
	EI			ESE			EI		
	*B*	*SE*	*t*	*B*	*SE*	*t*	*B*	*SE*	*t*
Male Students	0.138	0.056	2.486*	018	0.070	0.259	0.133	0.052	2.551*
Freshmen	0.794	0.058	13.750***	0.259	0.072	3.582***	0.722	0.056	12.979***
Second-year universities	1.491	0.072	20.840***	0.659	0.090	7.352***	1.308	0.074	17.627***
NP	0.235	0.035	6.750***	0.431	0.044	9.880***	0.115	0.039	2.975**
ESE							0.279	0.048	5.846***
*R* ^2^	0.756			0.515			0.786		
*F*	191.701***			65.497***			180.795***		

*, p < 0.05; **, p < 0.01; ***, p < 0.001.

*B is the unstandardized regression coefficient.*

*Gender and grade are dummy variables, in which male students are the experimental group and female students are the reference group, and freshmen and second-year universities form the experimental group and junior students are the reference group, respectively.*

*NP, Narcissistic Personality; ESE, Entrepreneurial Self-Efficacy; EI, Entrepreneurial Intention.*

## Discussion and Conclusion

### Theoretical Contributions

First of all, the results of this study found that the narcissistic personality of Chinese Beijing university students significantly positively affects entrepreneurial intentions. The result of this study is consistent with the findings of Mathieu and St-Jean (2013), O’Reilly et al. (2014), Hmieleski and Lerner (2016), Zia et al. (2020), and Cai et al. (2021). This study concludes that such college students like to try new things and take risks (Mathieu and St-Jean, 2013); narcissists are considerably more motivated to pursue their goals and thus show better decision-making abilities (Zia et al., 2020). Because college students with a high narcissistic personality care more about what others think of them, want to be praised and recognized, and believe that they outshine others in all aspects, they will prove themselves by starting their own businesses to sharpen their sense of superiority. Narcissistic personality can further entrepreneurial intention, that is, a high narcissistic personality can promote the formation of entrepreneurial intention among college students. However, the results of this study are inconsistent with Wu et al. (2019b). The reason may be that the narcissistic personality is divided into two parts, positive and negative (Wu et al., 2019b).

Immediately afterward, the results of this study revealed that the narcissistic personality of Chinese Beijing university students significantly positively affects entrepreneurial self-efficacy. The result of this study is consistent with the findings of Mathieu and St-Jean (2013) and Brookes (2015). This study concludes that college students with high narcissism aspire to improve themselves and are confident of their abilities (Forsyth et al., 2012). They believe they can perform better than others even when they fail in their efforts to start businesses (Mathieu and St-Jean, 2013). Furthermore, highly narcissistic college students pay close attention to themselves, overrate their importance, and believe that they are capable of doing anything. The recognition of themselves boosts their self-confidence. Therefore, entrepreneurial self-efficacy, that is, one’s degree of confidence in being able to successfully start a business, benefits entrepreneurial intentions. Hence, we argue that an increase in narcissistic personality increases entrepreneurial self-efficacy.

Secondly, this study found that the entrepreneurial self-efficacy of Chinese college students in Beijing significantly positively affects entrepreneurial intentions. The result of this study is consistent with the findings of Boyd and Vozikis (1994), Chen et al. (1998), Naktiyok et al. (2010), Pihie and Bagheri (2013), and Liu et al. (2019). This study concludes that entrepreneurial self-efficacy largely influences an individual’s intention to become an entrepreneur, their efforts to establish a new business, and their persistence in facing new challenges and changes in the entrepreneurial process (Trevelyan, 2011). In addition, college students with high entrepreneurial self-efficacy are confident of their entrepreneurial abilities and in their ability to succeed in starting businesses. The increase in self-efficacy, in turn, further helps to increase entrepreneurial intention.

Finally, this study found that the entrepreneurial self-efficacy of Chinese college students in Beijing plays a partial mediating role between narcissistic personality and entrepreneurial intention. The study results show that the higher the degree of college students’ narcissistic personality, the more it strengthens their entrepreneurial self-efficacy, which further helps to increase their entrepreneurial intention. The results are consistent with previous findings that entrepreneurial self-efficacy mediates the relationship between personality traits and entrepreneurial intentions (Zhao et al., 2005; Prabhu et al., 2012; St-Jean and Mathieu, 2015; Darmanto and Yuliari, 2018; Wu et al., 2019a), which confirms that entrepreneurial self-efficacy also mediates the relationship between narcissistic personality and entrepreneurial intentions. This study concludes that self-efficacy is closely related to the level of competence and personality traits of a person (Bandura, 1997). Therefore, narcissistic personality can be considered an individual characteristic and can affect entrepreneurial intentions through entrepreneurial self-efficacy. The higher the narcissistic personality of a college student, the better they think they are and the more confident they feel about themselves. This increases their entrepreneurial self-efficacy and they believe that they can establish their own businesses, which ultimately increases their entrepreneurial intention. Therefore, a narcissistic personality can indirectly affect entrepreneurial intention through entrepreneurial self-efficacy. In addition, this study suggests that entrepreneurial self-efficacy is an influential key mediating variable in entrepreneurship-related empirical studies. The finding provides empirical evidence for the use of self-efficacy theory in exploring college students’ entrepreneurial intention.

### Practical Implications

The study results provide higher vocational colleges with inspiration to carry out entrepreneurship education. First, when providing entrepreneurship education, higher vocational colleges should pay attention to students’ narcissistic personalities. The role of narcissistic personality in influencing entrepreneurial intention has been confirmed. Therefore, the institutions should improve the narcissistic personalities of college students by providing them opportunities to present themselves in teaching. Teachers and all walks of life should respect and recognize college students, etc. Entrepreneurship research groups can be formed to study effective methods to improve narcissistic personality. Besides, higher vocational colleges can make more efforts to help college students develop the skills and abilities needed for starting businesses and develop their ability to accept defeat. Furthermore, when assigning homework, teachers can appropriately assign some open-ended tasks to students for improving their open-mindedness and allowing them to report and summarize. Second, the impact of entrepreneurial self-efficacy on entrepreneurial intention has also been confirmed. Therefore, higher vocational colleges can conduct regular lectures of entrepreneurs and alumni who have succeeded in starting businesses and ask them to share their experiences. The institutions can make these speakers as role models for college students with entrepreneurial intention and strengthen their entrepreneurial self-efficacy. Furthermore, higher vocational colleges can provide these students with targeted training in the skills and abilities needed for starting businesses. When students have a positive and high entrepreneurial self-efficacy, that is, they adopt positive attitudes toward starting businesses and facing challenges, their development of entrepreneurial intention is enhanced.

### Limitations and Future Research Directions

This study has its limitations that inspire for future research. First, this study only considers the mechanisms influencing narcissistic personality, entrepreneurial self-efficacy, and entrepreneurial intention, but many factors affect entrepreneurial intention. For example, self-esteem has a significant impact on entrepreneurial intention in terms of an individual’s psychology (Laguna, 2013; Chen et al., 2016); social environment plays a mediation role in the relationship between self-esteem and entrepreneurial intention in terms of an external environment (Elali and Al-Yacoub, 2016; Hockerts, 2017). Therefore, we suggest that future researchers can explore other variables that affect entrepreneurial intentions, or add predictors of theory of planned behavior to their research models, such as attitude, performed behavioral control, behavior, etc (Ajzen and Madden, 1986). Moreover, this study was conducted only with college students from three higher vocational colleges in Beijing, China, and so, the inferences are relatively restricted. Expansion of the scope of respondents by investigating college students from other types of schools would further raise the value of relevant research.

## Data Availability Statement

The raw data supporting the conclusions of this article will be made available by the authors, without undue reservation.

## Ethics Statement

The studies involving human participants were reviewed and approved by Hainan Technology and Business College (HGS-2020-09). The participants provided their written informed consent to participate in this study. Written informed consent was obtained from the individual(s) for the publication of any potentially identifiable images or data included in this article.

## Author Contributions

S-YG designed the study, analyzed the data, and drafted the manuscript. JH assisted in analyzing and interpreting the data and participated in the revision of the manuscript. Both authors contributed to the study and approved the submitted version.

## Conflict of Interest

The authors declare that the research was conducted in the absence of any commercial or financial relationships that could be construed as a potential conflict of interest.

## Publisher’s Note

All claims expressed in this article are solely those of the authors and do not necessarily represent those of their affiliated organizations, or those of the publisher, the editors and the reviewers. Any product that may be evaluated in this article, or claim that may be made by its manufacturer, is not guaranteed or endorsed by the publisher.
