# Corrigendum: Effect of narcissistic personality on entrepreneurial intention among college students: mediation role of entrepreneurial self-efficacy

**DOI:** 10.3389/fpsyg.2023.1206340

**Published:** 2023-08-11

**Authors:** Sun-Yu Gao, Jianhao Huang

**Affiliations:** ^1^Dhurakij Pundit University, Bangkok, Thailand; ^2^Hainan Technology and Business College, Hainan, China

**Keywords:** entrepreneurial intention (EI), narcissistic personality, entrepreneurial self-efficacy (ESE), college students, mediation role

In the published article, there was an error regarding the affiliation of author “Sun-Yu Gao.” As well as having the affiliation “1. Dhurakij Pundit University, Bangkok, Thailand,” they should also have the affiliation, “2. Hainan Technology and Business College, Hainan, China.”

In addition, there was an error in the institution named as providing ethics approval for the study. It was listed as “Dhurakij Pundit University” but should be “Hainan Technology and Business College.” A correction has been made to **Methods**, “*Test Process*,” paragraph 1 as follows:

“This study was carried out in accordance with the commendations of Hainan Technology and Business College. Before the test, the teachers of the respondents were informed of the study intention. With their assistance, we asked students to fill out the questionnaire in a uniform manner. To ensure the objectivity and authenticity of the questionnaire data, we explained the confidentiality of the questionnaire results and the study purpose to the respondents.”

A correction has also been made to the “Ethics statement” to include the approving institution and approval number as follows:

## Ethics statement

The studies involving human participants were reviewed and approved by Hainan Technology and Business College (HGS-2020-09). The participants provided their written informed consent to participate in this study. Written informed consent was obtained from the individual(s) for the publication of any potentially identifiable images or data included in this article.

The authors apologize for these errors and state that this does not change the scientific conclusions of the article in any way. The original article has been updated.

